# Impregnation of Se_2_S_6_ into a Nitrogen- and Sulfur-Co-Doped Functional Metal Carbides and Nitrides for High-Performance Li-S Batteries

**DOI:** 10.3390/molecules30051070

**Published:** 2025-02-26

**Authors:** Lu Chen, Zhongyuan Zheng, Shuo Meng, Wenwei Wu, Weicheng Zhou, Shanshan Yang, Kexuan Liao, Yuanhui Zuo, Ting He

**Affiliations:** 1 School of Chemical Science and Engineering, Tongji University, Siping Road 1239, Shanghai 200092, China; 1810431@tongji.edu.cn (L.C.); 1811270@tongji.edu.cn (S.M.); 2333683@tongji.edu.cn (W.W.); 2411793@tongji.edu.cn (W.Z.); 2433931@tongji.edu.cn (S.Y.); 2 School of Chemistry and Chemical Engineering, Shanxi University, Wucheng Road 92, Taiyuan 030006, China; zzydeemail04@163.com; 3 Tsinghua Center for Green Chemical Engineering Electrification (CCEE), Beijing Key Laboratory of Green Chemical Reaction Engineering and Technology, Department of Chemical Engineering, Tsinghua University, Beijing 100084, China; liaokexuan@mail.tsinghua.edu.cn; 4 Institute of Wideband Semiconductor Materials and Devices, Fudan University, Ningbo 315327, China

**Keywords:** Se_2_S_6_, heteroatom dope, MXene, cathode, Li-S batteries

## Abstract

In this study, nitrogen- and sulfur-co-doped MXene (NS-MXene) was developed as a high-performance cathode material for lithium–sulfur (Li-S) batteries. Heterocyclic Se_2_S_6_ molecules were successfully confined within the NS-MXene structure using a simple melt impregnation method. The resulting NS-MXene exhibited a unique wrinkled morphology with a stable structure which facilitated rapid ion transport and provided a physical barrier to mitigate the shuttle effect of polysulfide. The introduction of nitrogen and sulfur heteroatoms into the MXene structure not only shifted the Ti d-band center towards the Fermi level but also significantly polarizes the MXene, enhancing the conversion kinetics and ion diffusion capability while preventing the accumulation of Li_2_S_6_. Additionally, the incorporation of Se and S in Se_2_S_6_ improved the conductivity compared to S alone, resulting in reduced polarization and enhanced electrical properties. Consequently, NS-MXene/Se_2_S_6_ exhibited excellent cycling stability, high reversible capacity, and reliable performance at high current densities and under extreme conditions, such as high sulfur loading and low electrolyte-to-sulfur ratios. This work presents a simple and effective strategy for designing heteroatom-doped MXene materials, offering promising potential for the development of high-performance, long-lasting Li-S batteries for practical applications.

## 1. Introduction

With the growing demand for advanced energy storage solutions, developing a new type of secondary battery with excellent energy conversion and storage capabilities has become the focus of research [1,2,3,4]. Among these, lithium-ion and lithium-air batteries have emerged as a leading technology due to their high energy density, safety, and long lifespans. However, despite these advantages, the energy density of commercial lithium-ion and lithium-air batteries remains limited, rarely exceeding 300 Wh kg^−1^, thus failing to meet the stringent performance requirements across various applications [5,6].

Lithium–sulfur (Li-S) batteries has been regarded as one of the most promising candidates for the next-generation battery technology due to that high theoretical energy density of 2600 Wh kg^−1^, far surpassing that of conventional lithium-ion and lithium-air batteries. Moreover, the abundant and environmentally friendly nature of sulfur enhances the appeal of Li-S batteries as a next-generation energy storage solution. As large-scale electrochemical energy storage applications, such as electric vehicles and energy storage stations, gain momentum, Li-S batteries have garnered significant attention in recent years [7,8,9,10,11,12,13,14,15,16,17,18,19,20]. However, the commercialization of Li-S batteries remains hindered by several challenges. Notably, the poor electrical conductivity of sulfur and lithium sulfide (Li_2_S), coupled with the extreme volumetric expansion during the full lithiation of sulfur into Li_2_S, presents significant obstacles. What is more, the shuttle effect is largely attributed to the dissolution of intermediate lithium polysulfide (LiPS) species into the organic electrolyte. Specifically, it refers to the irreversible migration of polysulfides between the cathode and anode during charge/discharge cycles and leads to capacity decay. The shuttle effect continues to pose a substantial barrier to the practical application of Li-S batteries [8,17,18,19,20].

To mitigate this issue, a key approach has been the introduction of host materials that facilitate robust polysulfide adsorption and catalytic conversion, which has shown promise in reducing the detrimental effects of the shuttle phenomenon [10,21,22]. The enhancement of electronic conductivity in sulfur-based electrodes is vital for improving the performance of lithium–sulfur (Li-S) batteries. Traditional approaches typically involve incorporating highly conductive carbon materials, such as acetylene black, carbon black, and graphene, into cathode composites [23,24,25]. While these materials effectively improve conductivity, their weak interaction with the polar lithium polysulfides leads to the diffusion of polysulfides into the electrolyte during charge and discharge cycles, which adversely affects battery performance.

To overcome this limitation, heteroatom doping has emerged as a promising strategy for modifying the carbon-based conductive network, enhancing its affinity for lithium polysulfides and alleviating the shuttle effect. Previous research has demonstrated the effectiveness of heteroatom-doped carbon materials in stabilizing lithium polysulfides [26,27,28]. This approach involves introducing heteroatoms such as nitrogen (N), oxygen (O), and sulfur (S) into carbon materials. The presence of these dopants significantly polarizes the carbon matrix, thereby improving its ability to absorb the electrolyte and anchor lithium polysulfides through coordination bonds between the doped atoms and lithium ions.

Two-dimensional transition metal carbides and nitrides (MXenes), graphene-like layered materials with abundant surface functional groups, have attracted considerable attention in the energy field [29,30]. These materials are valued for their unique 2D structure, high electrical conductivity, and excellent surface chemistry. The high conductivity of MXenes effectively addresses the poor intrinsic conductivity of sulfur [19,20,28]. Moreover, the metal atoms and surface functional groups within MXenes enable strong interactions with polysulfides, effectively suppressing their dissolution and mitigating the shuttle effect. Additionally, MXenes have been shown to accelerate the redox kinetics of intermediate products in Li-S batteries, making them an ideal candidate for enhancing the performance of such systems.

Selenium, a congener of sulfur, exhibits significantly higher electronic conductivity (1 × 10^−3^ S m^−1^) and a comparable theoretical volumetric capacity density (3240 mAh cm^−3^). As a result, mixed Se-S cathode materials have been proposed as promising candidates for high-energy lithium batteries, offering advanced overall performance in recent years [19,31,32]. In this regard, Se_2_S_6_ was chosen due to its high conductivity and compatibility with the MXene framework. Its incorporation facilitates enhanced electron transport and reduced polarization, thereby working synergistically with MXene to improve polysulfide adsorption and overall battery performance.

In this work, a nitrogen- and sulfur-co-doped MXene (NS-MXene) with a distinctive capsule-like structure was successfully synthesized using a simple thiourea-induced pyrolysis method. The NS-MXene sheets form an interconnected conductive network, facilitating rapid electron and ion migration. The expanded 2D structure, with increased interlayer spacing, acts as a physical barrier that restricts the movement of polysulfides but does not impede the migration of Li^+^ ions, owing to the enlarged ion transport pathways and enhanced ion diffusion kinetics. Furthermore, the incorporation of selenium enhances electron transfer during the electrochemical reaction and reduces polarization effects, excessive polarization is detrimental to battery performance, and the controlled introduction of selenium helps mitigate polarization effects, thereby enhancing efficiency and making Se_2_S_6_ more electrochemically efficient than sulfur. Consequently, the assembled cell exhibited outstanding long-term cycling performance, with an initial capacity of 974 mAh g^−1^ and a low capacity decay rate of 0.061% per cycle at 0.5 A g^−1^. The cell also demonstrated excellent rate performance, retaining 315 mAh g^−1^ at a high current density of 4 A g^−1^ and stable cycling performance with high loading (6.2 mg cm^−2^, E/S = 8.2 μL mg^−1^). These results underscore the potential of this material for large-scale production and practical applications in lithium–sulfur batteries.

## 2. Result and Discussion

**Figure 1:**
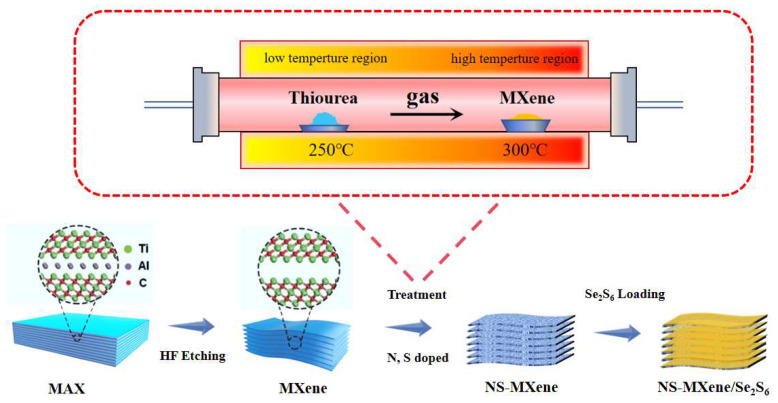
Synthesis diagram of NS-MXene/Se_2_S_6_ cathode material.

The preparation process of NS-MXene/Se_2_S_6_ is schematically illustrated in Figure 1. The accordion-like MXene was first obtained by etching MAX-phase Ti_3_AlC_2_ with HF. The resulting MXene was placed in the high-temperature section of a closed tube furnace at 300 °C, while thiourea was introduced into the low-temperature section at 250 °C. During the heat treatment, thiourea decomposed, releasing hydrogen sulfide and ammonia. These gaseous products interacted with the MXene in the high-temperature section, replacing a portion of the carbon atoms in MXene, thus yielding NS-MXene. In addition, Se_2_S_6_ solid solutions were prepared by comelting commercial selenium and sulfur powders. The color of the sulfur powder is yellow, the selenium powder is gray, and the prepared Se_2_S_6_ powder is orange (Figure S1). Following this, a mixture of NS-MXene and Se_2_S_6_ powder was subjected to further heat treatment, allowing Se_2_S_6_ to be fully incorporated into the interlayers of MXene, resulting in the formation of the NS-MXene/Se_2_S_6_ composite.

**Figure 2:**
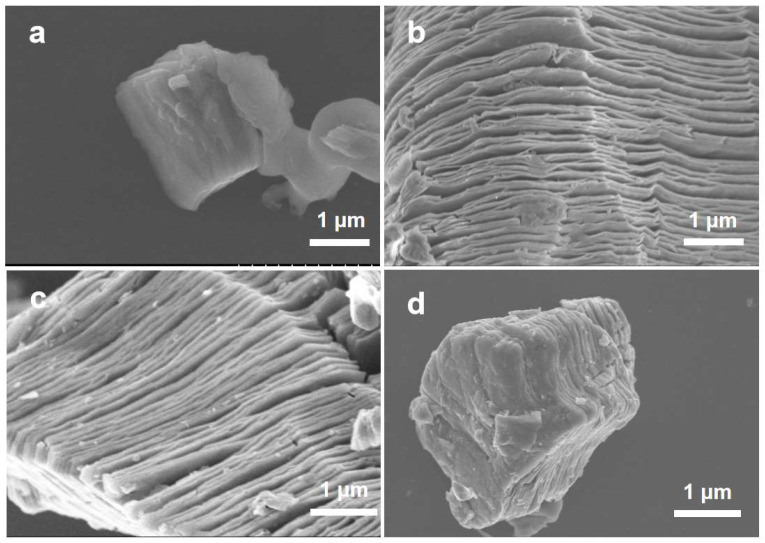
SEM images of (**a**) MAX-phase Ti_3_AlC_2_ (**b**) MXene (**c**) NS-MXene (**d**) NS-MXene/Se_2_S_6_.

The morphologies of the MAX-phase Ti_3_AlC_2_, MXene, NS-MXene, and NS-MXene/Se_2_S_6_ samples were characterized by scanning electron microscopy (SEM) and are shown in Figure 2. As illustrated in Figure 2a,b, HF etching effectively removes the Al layers from the MAX-phase Ti_3_AlC_2_, leaving behind well-stacked nanosheets that form the accordion-like structure of MXene. In Figure 2c, the SEM images of NS-MXene reveal similarly well-stacked nanosheets, indicating that the primary structure of MXene remains largely unchanged under the high-temperature conditions of ammonia and hydrogen sulfide treatment. Figure 2d shows the SEM images of NS-MXene/Se_2_S_6_, where it is evident that the gaps between the layers of NS-MXene are uniformly filled with Se_2_S_6_, confirming the successful preparation of the NS-MXene/Se_2_S_6_ composite.

**Figure 3:**
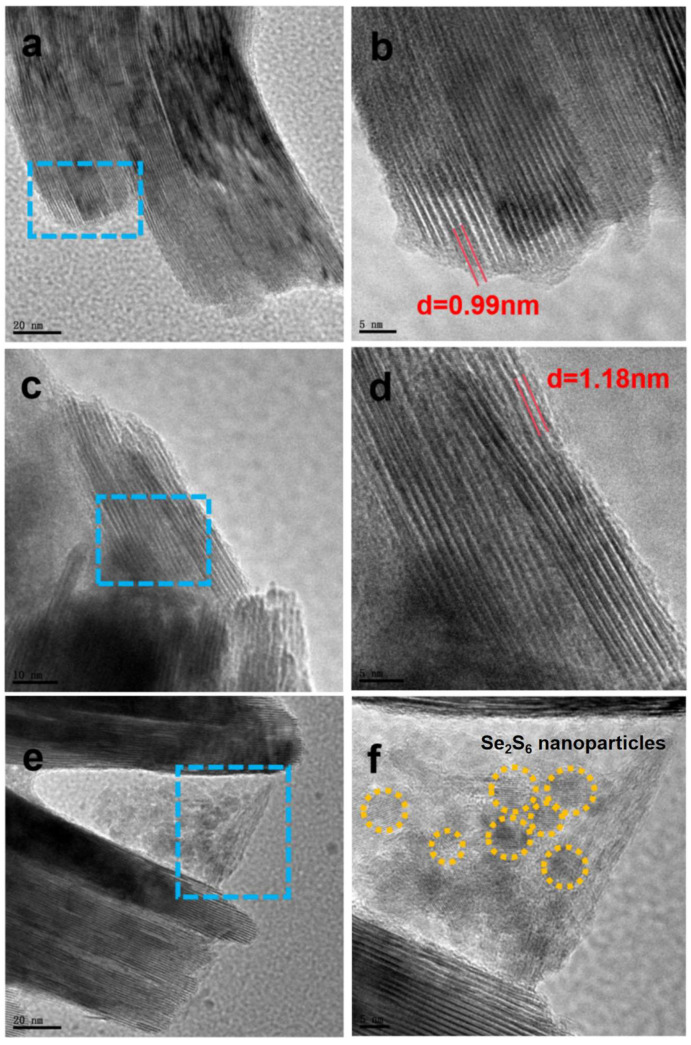
TEM and HRTEM images of (**a**,**b**) MXene, (**c**,**d**) NS-MXene, (**e**,**f**) NS-MXene/Se_2_S_6_.

To further investigate the microscopic structure, transmission electron microscopy (TEM) and high-resolution TEM (HRTEM) were employed to analyze the detailed structures of MXene, NS-MXene, and NS-MXene/Se_2_S_6_. In Figure 3a, MXene exhibits a bulk microscale feature with a multilayered structure, further confirming the successful etching of the Al layer from the MAX-phase. The clear gaps between the layers of MXene are also observable. HRTEM images of MXene, shown in Figure 3b, reveal a multilayer crystal structure with an interlayer spacing of 0.99 nm, corresponding to the (002) plane of MXene. Figure 3c,d show that the multilayered crystal structure of NS-MXene is well-preserved, with an expanded interlayer spacing of 1.18 nm, confirming that the co-doping of nitrogen and sulfur atoms in NS-MXene led to this expansion. Furthermore, the high-resolution TEM images of NS-MXene/Se_2_S_6_ are shown in Figure 3e,f, and the round-shaped Se_2_S_6_ nanoparticles with a size of 3–8 nm could be clearly detected in the (103) lattice plane of the MXene, which illustrates that Se_2_S_6_ is loaded in the open channels between the layers of NS-MXene.

**Figure 4:**
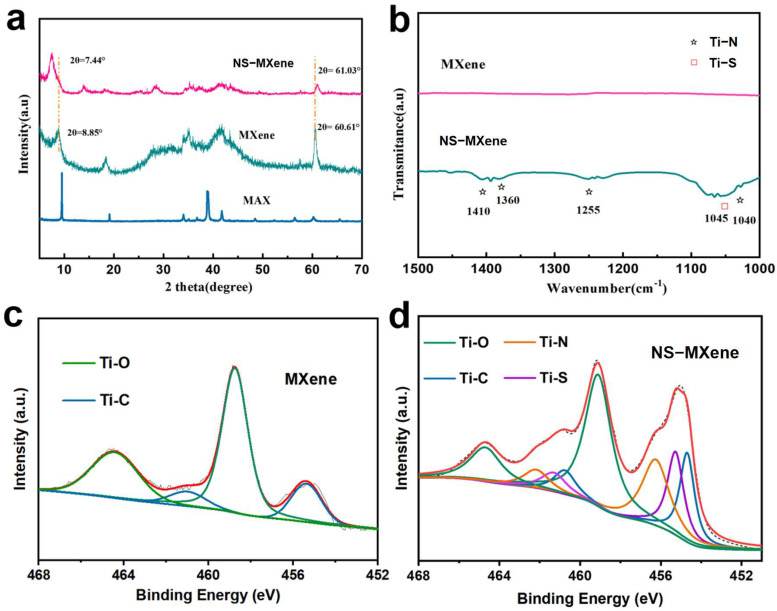
(**a**) XRD patterns of Ti_3_AlC_2_ MAX-phase, MXene and NS-MXene samples. (**b**) FTIR spectrum of MXene and NS-MXene sample. Ti 2p spectrum of the (**c**) MXene and (**d**) NS-MXene composites.

X-ray diffraction (XRD) was further employed to investigate the phase structures of MAX-phase Ti_3_AlC_2_, MXene, and NS-MXene. As shown in Figure 4a, the sharp peaks in the XRD spectrum of Ti_3_AlC_2_ indicate its high crystallinity and purity, with diffraction peaks aligning with the standard pattern of JCPDS52-0875. Upon comparison with the pristine Ti_3_AlC_2_ phase, the disappearance of the strongest (104) peak and the appearance of a broader (002) peak, the signal confirms the successful etching of the Al layer from the Ti_3_AlC_2_ phase during HF treatment. The characteristic (002) peak of MXene appears at 8.85°, corresponding to an interlayer distance of 0.99 nm, which is consistent with the HRTEM results. Furthermore, the XRD patterns of MXene treated with ammonia and hydrogen sulfide reveal a significant shift in the (002) diffraction peak to 7.44°, which corresponds to an enhanced interlayer spacing of 1.18 nm, in agreement with the interlayer distance observed in the HRTEM images of NS-MXene. In addition, the shift in the peak positions between MXene and NS-MXene can be attributed to the incorporation of N and S heteroatoms into the MXene structure; such incorporation not only enlarges the interlayer spacing but may also involve an electron-donating mechanism. This mechanism could cause the Ti d-band center to shift toward the Fermi level, thereby altering the electron cloud distribution within the crystal structure.

Additionally, Fourier transform infrared spectroscopy (FT-IR) was employed to further characterize the Ti-N and Ti-S bonds in NS-MXene. As shown in Figure 4b, several characteristic FTIR peaks in the NS-MXene spectrum, compared to that of MXene, are observed. The characteristic peaks located at 1410, 1360, 1255, and 1045 cm^−1^ are attributed to the vibrations of the Ti-N bond, while the peak around 1040 cm^−1^ corresponds to the vibrations of the Ti-S bond [28,33]. These results provide further evidence that nitrogen and sulfur heteroatoms successfully replaced part of the carbon atoms in MXene, confirming the formation of NS-MXene.

X-ray photoelectron spectroscopy (XPS) was then performed to examine the elemental valence states and surface chemical bonding of MXene and NS-MXene. The XPS spectra of high-resolution Ti 2p for MXene and NS-MXene are shown in Figure 4c,d. In Figure 4c, the binding energy peaks at 458.7 and 464.5 eV correspond to Ti-O bonds, while peaks at 461.0 and 455.4 eV are attributed to Ti-C bonds [29,30]. In the case of NS-MXene composites, the XPS spectra in Figure 4d reveal the presence of Ti-N and Ti-S bonds in addition to the Ti-O and Ti-C bonds. Specifically, peaks at 456.3 and 462.2 eV are attributed to Ti-N bonds, while peaks at 461.3 and 455.3 eV correspond to Ti-S bonds [12,28]. These XPS results confirm that nitrogen and sulfur atoms have successfully replaced the carbon atoms in MXene, resulting in the formation of NS-MXene.

**Figure 5:**
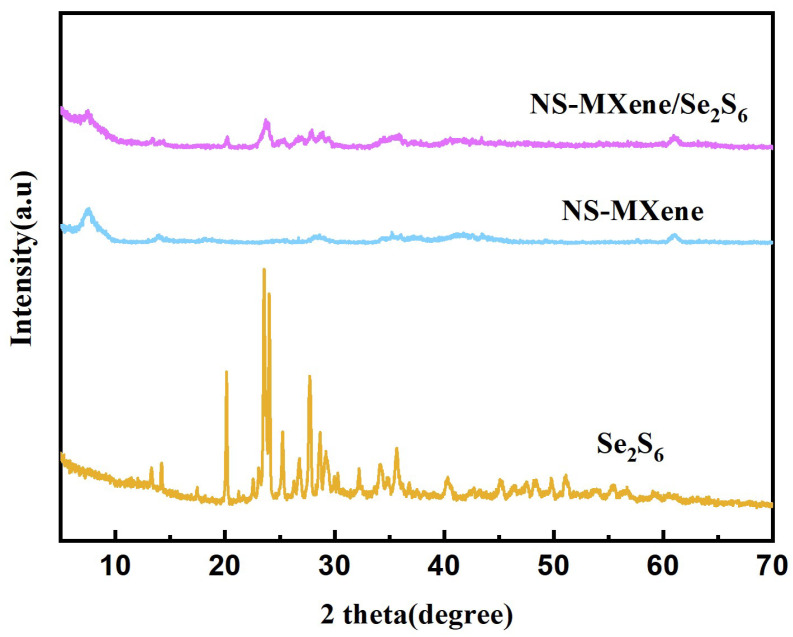
XRD patterns of Se_2_S_6_, NS-MXene/Se_2_S_6_, and NS-MXene samples.

The X-ray diffraction (XRD) patterns of NS-MXene/Se_2_S_6_ composites were measured and are presented in Figure 5. The XRD patterns clearly show the characteristic peaks of Se_2_S_6_ and the as-prepared NS-MXene, respectively. EDS patterns of NS-MXene/Se_2_S_6_ (Figure S2) further verified the removal of aluminum from the MAX phase. In addition, Figure S3 show that the XRD patterns of the Se, S, and Se_2_S_6_ samples are consistent with the previous literature [17,31]. In addition, the SEM images and EDS patterns of Se_2_S_6_ (Figure S4) further prove the successful preparation of Se_2_S_6_. Moreover, the TGA curve of NS-MXene/Se_2_S_6_ (Figure S5) calculates that the content of Se_2_S_6_ in NS-MXene/Se_2_S_6_ is 66.7%. Notably, no peaks corresponding to the anatase or rutile phases of TiO_2_ were observed in the XRD pattern of NS-MXene, further confirming the successful preparation of NS-MXene without the formation of TiO_2_ phases.

**Figure 6:**
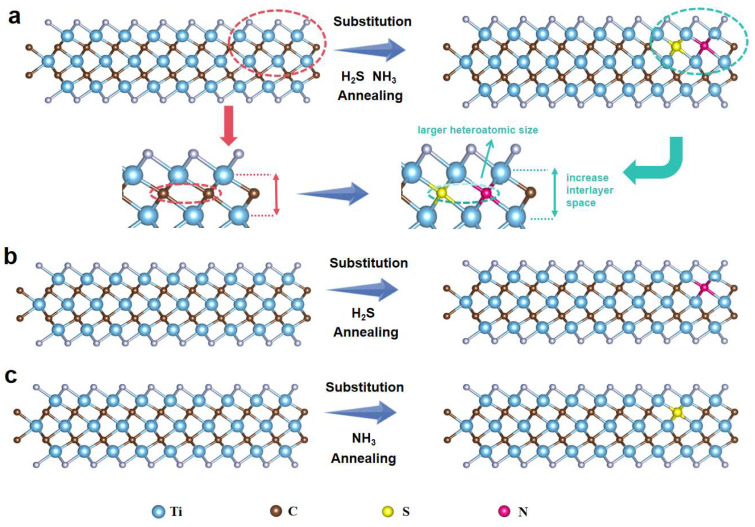
Schematic illustration of the MXene doped with (**a**) N,S atoms, (**b**) N atoms, (**c**) S atoms.

Figure 6 illustrates a schematic representation of the heteroatom-doped MXene structure, where nitrogen (N) and sulfur (S) atoms simultaneously replace carbon (C) atoms, as well as scenarios where N atoms replace C and S atoms replace C separately. Due to the larger atomic sizes of N and S compared to C, the interlayer spacing of the MXene structure is expanded. The introduction of N and S atoms also influences the neighboring carbon atoms, further enhancing the expansion of the layer spacing. In addition, the introduction of nitrogen and sulfur heteroatoms into the MXene structure not only shifted the Ti d-band center towards the Fermi level but also significantly polarized the MXene, enhancing the conversion kinetics and ion diffusion capability while preventing the accumulation of Li_2_S_6_.

**Figure 7:**
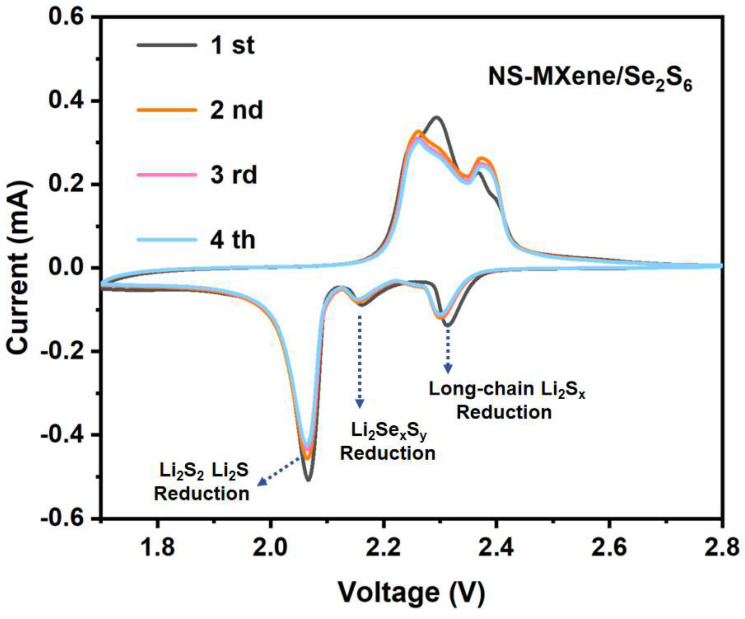
CV curves of cells with NS-MXene/Se_2_S_6_ as cathodes at a scan rate of 0.1 mV S^−1^.

Figure 7 presents the cyclic voltammetry (CV) curves of NS-MXene/Se_2_S_6_ cathodes tested at a scan rate of 0.1 mV s^−1^ within the voltage range of 1.7 to 2.8 V. Three distinct cathodic reduction peaks, along with two anodic oxidation peaks, are observed in the CV curve of NS-MXene/Se_2_S_6_. These peaks correspond to the multistep lithiation processes of Se_2_S_6_ and the subsequent delithiation and conversion of Li_2_S/Li_2_Se, which is consistent with previous reports in the literature [17,31]. The reduction peaks at 2.32 V and 2.07 V are attributed to the conversion of high-order lithium polysulfides to low-order polysulfides and then to solid-state Li_2_S [12,13,14,15,18]. Additionally, the reduction peak at 2.17 V can be ascribed to the formation of polysulfoselenide species (Li_2_Se_x_S_y_, where 4 ≤ x + y < 8) [17]. For comparison, the CV curves of cells with NS-MXene/S are shown in Figure S6, and the reduction peak at 2.17 V is not observed. Moreover, as shown in Figure S7, the CV curve of NS-MXene/Se_2_S_6_ exhibits a positive shift in the cathodic peak and a negative shift in the anodic peak along with significantly higher peak intensities compared to the NS-MXene/Se_2_S_6_ cathode. This indicates a substantial enhancement in the activity and reaction kinetics for polysulfide conversion, which can be attributed to the presence of N and S atoms in the co-doping process. In addition, NS-MXene/Se_2_S_6_ exhibits a noticeable shift in oxidation peaks between the first cycle and the subsequent cycles; which is due to that the incorporation of Se_2_S_6_ in NS-MXene/Se_2_S_6_ leads to an initial activation process that alters the oxidation peak in the first cycle before stabilizing in subsequent cycles.

**Figure 8:**
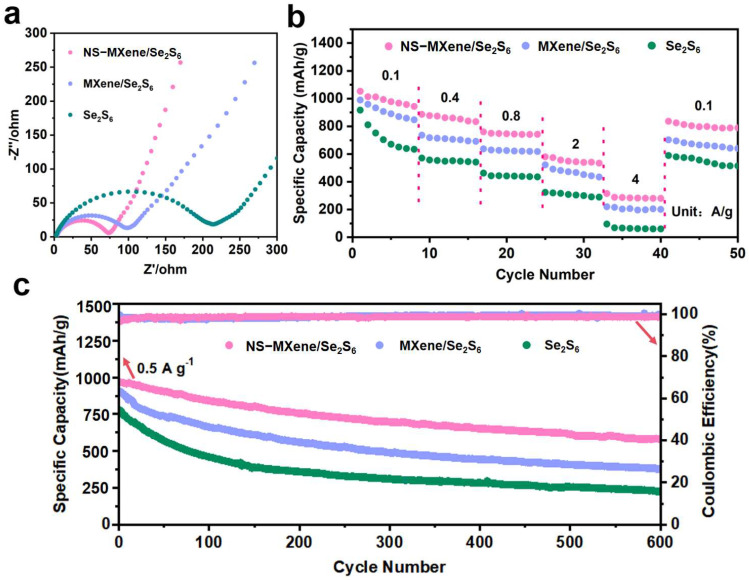
(**a**) The Nyquist plots, (**b**) cycle performance, and (**c**) rate performance of Se_2_S_6_, MXene/Se_2_S_6_ and NS-MXene/Se_2_S_6_ cathodes.

The above results demonstrate that NS-MXene exhibits superior adsorption effects on polysulfides, a larger interlayer spacing, and a greater sulfur storage capacity compared to MXene. To further assess the electrochemical performance of NS-MXene/Se_2_S_6_ electrodes and MXene/S electrodes, the cell assembly is depicted in Figure S8. Before tests, the theoretical gravimetric capacity of the S, Se and Se_2_S_6_ were calculated (Figure S9). The cycling performance at various current rates is shown in Figure 8b. The NS-MXene/Se_2_S_6_ cathode delivers a reversible capacity of 1053, 886, 760, 580, and 315 mAh g^−1^ at current rates of 0.1, 0.4, 0.8, 2, and 4 A g^−1^, respectively. Upon returning the current to 0.1 A g^−1^, the NS-MXene/Se_2_S_6_ cathode retains a capacity of 835 mAh g^−1^. In comparison, the cells with MXene/Se_2_S_6_ show average capacities of 1001.2, 711.4, 638.6, 515.6, and 231.1 mAh g^−1^ at current rates of 0.1, 0.4, 0.8, 2, and 4 A g^−1^, respectively. When returning the current to 0.1 A g^−1^, the MXene/Se_2_S_6_ cathode only retains a capacity of 710.4 mAh g^−1^. In addition, the Se_2_S_6_ cathodes exhibit an even poorer rate performance than MXene/Se_2_S_6_ and NS-MXene/Se_2_S_6_. The cycle performance of Se_2_S_6_, MXene/Se_2_S_6,_ and NS-MXene/Se_2_S_6_ was shown in Figure 8c, and the NS-MXene/Se_2_S_6_ cathode delivers a capacity of 974.2 mAh g^−1^ in the first cycle and retains 614.8 mAh g^−1^ after 600 cycles, demonstrating a high capacity retention of 62.8%. In contrast, the MXene/Se_2_S_6_ cathode delivers 920 mAh g^−1^ in the first cycle at 0.5 A g^−1^ after 600 cycles, resulting in a low capacity retention of 40.4%. Furthermore, the Se_2_S_6_ cathodes exhibit even poorer cycling performance, delivering only 760 mAh g^−1^ in the first cycle at 0.5 A g^−1^, with capacity retentions of just 31.5% after 600 cycles. In addition, a near-100% coulombic efficiency has been found in this work, which further illustrates the excellent reversibility and minimal side reactions for NS-MXene/Se_2_S_6_.

**Figure 9:**
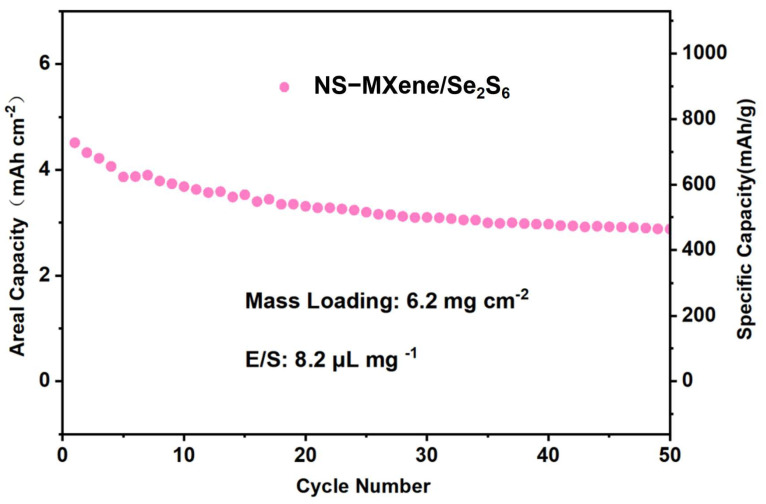
Cycling performance of the NS-MXene/Se_2_S_6_ for specific capacity and areal capacity.

To evaluate the practical application potential of NS-MXene/Se_2_S_6_, battery performance tests with high mass loading and high areal capacity (*C*_areal_) were conducted. *C*_areal_ is a crucial performance metric for batteries, directly influencing actual battery size, pack-level energy density, and manufacturing costs [34,35]. As shown in Figure 9, the NS-MXene/Se_2_S_6_ electrode with an areal loading of 6.2 mg cm^−2^ delivers a gravimetric capacity of 721.4 mAh g^−1^, corresponding to an areal capacity of 4.48 mAh cm^−2^, which greatly exceeds the benchmark value of 4 mAh cm^−2^ for commercial lithium-ion batteries (LIBs). After 50 cycles, the gravimetric capacity and areal capacity of the cathode remain at 471.2 mAh g^−1^ and 2.93 mAh cm^−2^, respectively, with a cycle retention rate of 65.3%. It is noted that the ultrathick electrodes with high loadings of the NS-MXene/Se_2_S_6_ were fabricated by mechanically pressing a self-standing NS-MXene/Se_2_S_6_ film into the Ti-network (Figure S10).

## 3. Method

### 3.1. Experimental Section

All the reagents used in this work were analytical grade and employed as purchased without further purification. Ti_3_AlC_2_ powders (99.8% weight percentage) supplied by 11 Technology Co., Beijing, China. Selenium and sulfur powders were purchased from Alading (Shanghai, China). Ethanol (99%) was purchased from Adamas (Shanghai, China).

### 3.2. Preparation of MXene

MXene was prepared by HF treatment of Ti_3_AlC_2_. Typically, 1 g of Ti_3_AlC_2_ powder was slowly introduced into the 10 mL HF solution. The suspension was kept at 40 °C for 24 h under stirring. Then, the mixture was centrifuged and washed with deionized water several times until PH = 7. MXene sample was washed three times with ethanol and DI water, dried under vacuum at 80 °C and prepared for use.

### 3.3. Preparation of NS-MXene

A sample of 200 mg of as-prepared MXene was placed in the low-temperature section of a closed tube furnace at 250 °C, 500 mg of thiourea was introduced into the high-temperature section at 300 °C after heat treatment at Ar atmosphere for 3 h to obtain the final product (NS-MXene).

### 3.4. Synthesis of the Se_2_S_6_

The Se_2_S_6_ solid solutions were prepared by comelting commercial selenium and sulfur powders. In a typical synthesis, sulfur and selenium powders with a designed molar ratio were mixed and sealed in a glass tube under vacuum. The sealed glass tube was heated up to 480 °C with a heating rate of 5 °C min^−1^ in a quartz tube and then held at 480 °C for 8 h before cooling back to room temperature naturally.

### 3.5. Synthesis of the NS-MXene/Se_2_S_6_

The NS-MXene/Se_2_S_6_ was prepared by heat treating NS-MXene and Se_2_S_6_ powders at 180 °C. In a typical synthesis, NS-MXene and Se_2_S_6_ powders with a designed molar ratio (1:2) were mixed for 30 min and then sealed in a glass tube under vacuum. The sealed glass tube was heated up to 180 °C with a heating rate of 3 °C min^−1^ in a quartz tube and then held for 2 h before cooling back to room temperature naturally. After being cooled down to room temperature, the NS-MXene/Se_2_S_6_ was successfully collected.

### 3.6. Material Characterizations

The morphology of the as-prepared samples was characterized by scanning electron microscopy (SEM), SEM images were acquired with (JEOL, JSM-7900F, Akishima, Japan). Transmission electron microscopy (TEM) and high-resolution transmission electron microscope (HRTEM) observations were conducted with a (JEOL, JEM-2011, Akishima, Japan) microscope operated at 200 kV and a microscope (JEOL, JEM-ARM200F, Akishima, Japan) equipped with an energy-dispersive spectrometer (EDS). X-ray diffraction patterns (XRD) were obtained by using a X-ray diffractometer (D8 advance, Bruke, Germany) with a Cu Kα radiation source (λ = 0.15418 nm). An X-ray photoelectron spectroscopy (XPS) investigation was conducted on a ESCA system (Perkin Elmer, PHI-5000C, Waltham, MA, USA) with Mg Kα radiation and the C1s peak at 284.6 eV as the internal standard.

### 3.7. Electrochemical Measurements

The CR2016 coin-type cells were assembled in an Ar-filled glovebox with a Celgard 2500 membrane as the separator, and lithium metal as the counter electrode. The thickness of the lithium foil is around 0.6 mm. The Li metal anode was used directly without any pretreatment. To prepare the electrodes, active materials, namely Ketjen black (ECP-600JD) and LA133 binder, were mixed with a mass ratio of 7:2:1 to form a slurry. The slurries were also coated onto a C-coated Al foil current collector and dried in a vacuum at 60 °C overnight. The Al foil was then punched into 14 mm disks. The high-loading electrode was fabricated by compressing a mixture of the NS-MXene/Se_2_S_6_ powder, Ketjen black (KB), and poly(vinylidene difluoride) (PTFE, 10 wt% aqueous solution) binder in a mass ratio of 7:2:1 on the titanium mesh followed by drying at 60 °C for 12 h. The gravimetric capacity and areal capacity are all calculated based on all the mass of active materials Se_2_S_6_. The areal capacity (mAh cm^−2^) is the capacity (mAh) per square centimeter (cm^2^). It can also be calculated as gravimetric capacity (mAh g^−1^) × mass loading of active material (g cm^−2^). The area of the round electrode could be calculated with the diameter. The electrolyte was 1 M Lithium bis (trifluoromethane sulfonyl) imide (LiTFSI) in a mixed solvent of 1,2-dimethoxyethane (DME) and 1,3-dioxolane (DOL) (1:1 by volume) with 1 wt % LiNO_3_ as the electrolyte additive. The galvanostatic charge/discharge measurement was conducted on a LAND CT2001A system within a voltage window of 1.7–2.8 V.

## 4. Conclusions

In summary, the N, S heteroatom co-doped functionalized MXene was constructed through a facile method, and the mixed Se-S materials in the form of heterocyclic Se_2_S_6_ molecules have been homogeneously confined within the NS-MXene by a facile melt impregnation route, which was used as high-performance Li-S battery cathode material. The distinctive morphology with a high stable structure can assure fast ion transport channels, and the cross-linked 2D stacking layer serves as a physical barrier for polysulfides to relieve shuttle effects. In addition, N, S heteroatom co-doping process significantly polarizes the MXene, boosting the conversion kinetics and ion diffusion capability and further preventing the accumulation of polysulfides. Accordingly, the Li-S battery with NS-MXene/Se_2_S_6_ expressed excellent cycling stability and desirable reversible capacity under high current densities and high capacity and reliable cycling under extreme conditions of high loading and a lean E/S ratio. This work presents a facile and effective strategy for developing a porous heteroatom co-doped MXene with a long lifespan and high-performance Li-S battery for potential practical applications.

## Data Availability

The data that support the findings of this study are available from the corresponding author upon reasonable request.

## Supplementary Materials

The following supporting information can be downloaded at: https://www.mdpi.com/article/10.3390/molecules30051070/s1, Figure S1: The optical photo of the S, Se, Se_2_S_6_; Figure S2: EDS patterns of NS-MXene/Se_2_S_6_; Figure S3: XRD patterns of Se, S, Se_2_S_6_ samples; Figure S4: (a) SEM images and (b) EDS patterns of Se_2_S_6_; Figure S5: The TGA curve of NS-MXene/Se_2_S_6_; Figure S6: CV curves of cells with NS-MXene/S as cathodes at a scan rate of 0.1 mV S^−1^; Figure S7: CV curves of cells with NS-MXene/S and NS-MXene/Se_2_S_6_ as cathodes for the second cycle at a scan rate of 0.1 mV s^−1^; Figure S8: The structure diagram of Assembly cell; Figure S9: Theoretical specific capacity of the Se, S and Se_2_S_6_; Figure S10: The digital photos of (a) Ti network (b) a self-standing NS-MXene/Se_2_S_6_ film (c) and Ti network-supported NS-MXene/Se_2_S_6_ electrode with high areal loading fabricated by mechanically pressing the NS-MXene/Se_2_S_6_ film onto current collector of Ti network.
