# Supplementary material for: Impregnation of Se2S6 into a Nitrogen- and Sulfur-Co-Doped Functional Metal Carbides and Nitrides for High-Performance Li-S Batteries
Source: Molecules. 2025 Feb 26;30(5):1070. doi: 10.3390/molecules30051070 (PMC11901854; doi:10.3390/molecules30051070)
Supplement: Supplementary file 1 [file molecules-30-01070-s001.zip › molecules-3470265-supplementary.pdf]

Supporting Information

Impregnation of Se_2S_6 into a Nitrogen and Sulfur Co-Doped Functional MXene for High-Performance Li-S Batteries

Lu Chen, Zhongyuan Zheng, Shuo Meng, Wenwei Wu, Weicheng Zhou, Shanshan Yang, Kexuan Liao, Yuanhui Zuo, Ting He**

*Dr. L. Chen, Dr. S. Meng, W. Wu, W. Zhou, S. Yang, Prof. T. He**

School of Chemical Science and Engineering, Tongji University, Siping Road 1239, Shanghai 200092, P. R. China

Z. Zheng

School of Chemistry and Chemical Engineering, Shanxi University, Wucheng Road 92, Shanxi 030006, P. R. China

Dr. K. Liao

Tsinghua Center for Green Chemical Engineering Electrification (CCEE), Beijing Key Laboratory of Green Chemical Reaction Engineering and Technology, Department of Chemical Engineering, Tsinghua University, Beijing 100084, China

Dr. Y. Zuo

Research Institute of Fudan University in Ningbo, Zhejiang 315327, China

Corresponding Authors: zuo_yuanhui@fudan.edu.cn, heting@tongji.edu.cn

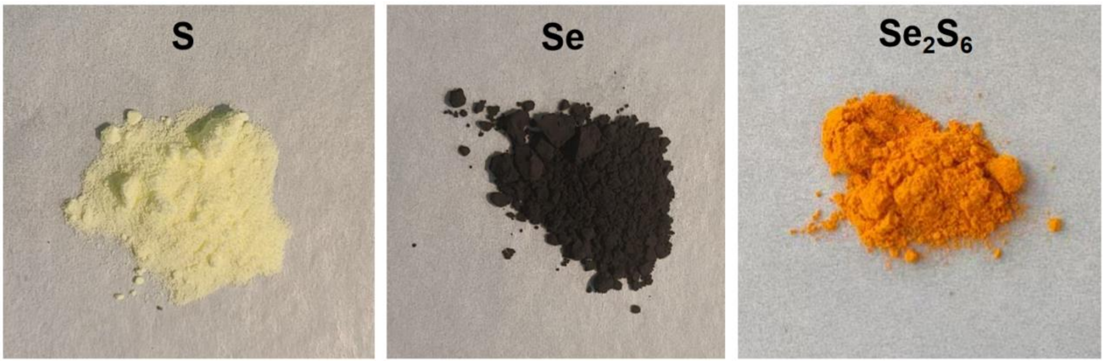

Figure S1. The optical photo of the S, Se, Se_2S_6.

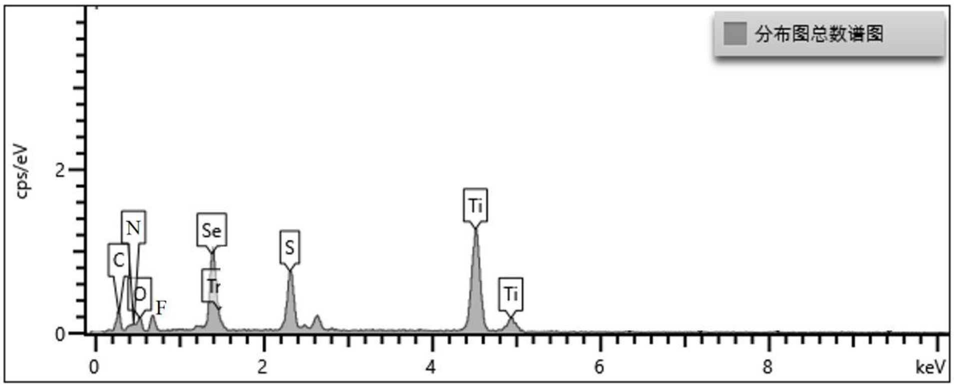

Figure S2. EDS patterns of NS-MXene/Se_2S_6.

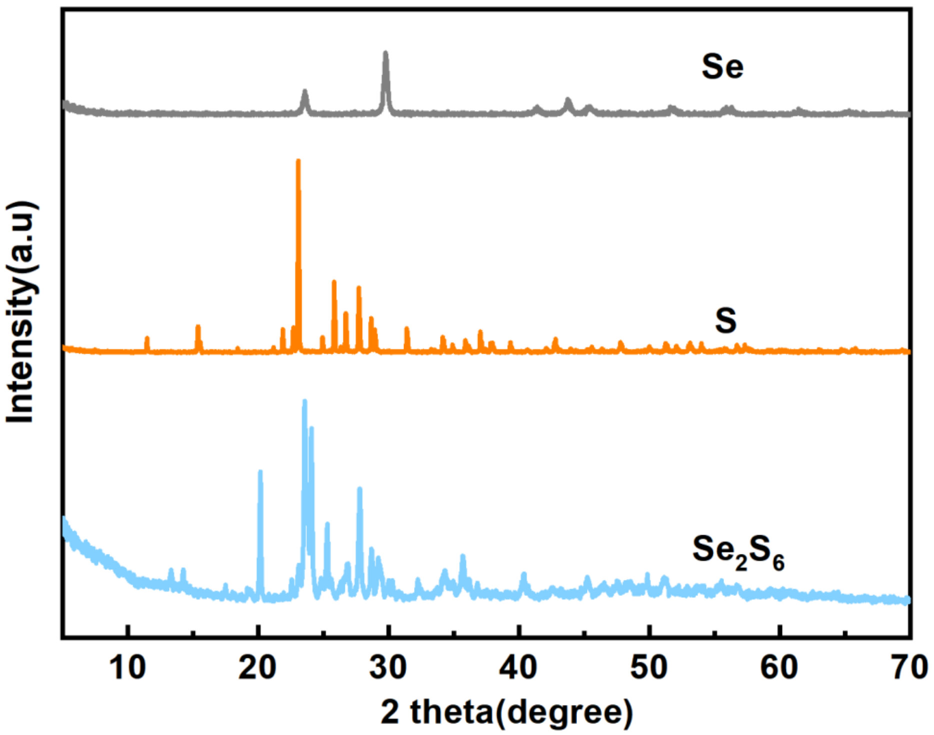

Figure S3. XRD patterns of Se, S, Se_2S_6 samples.

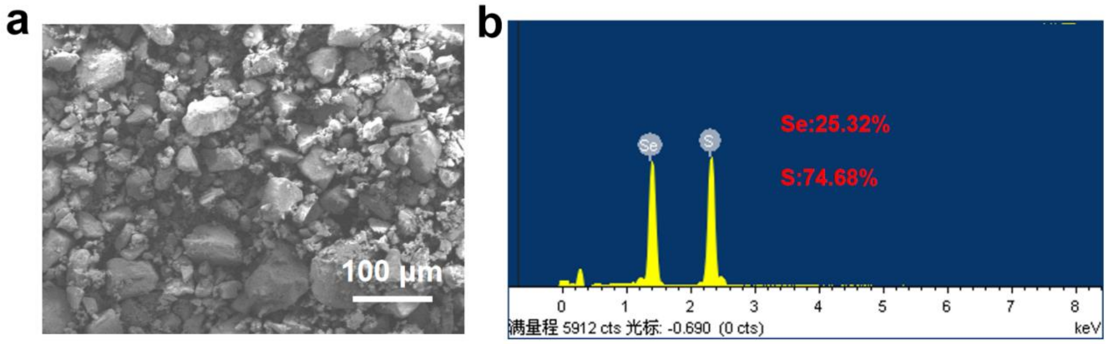

Figure S4. (a) SEM images and (b) EDS patterns of Se_2S_6.

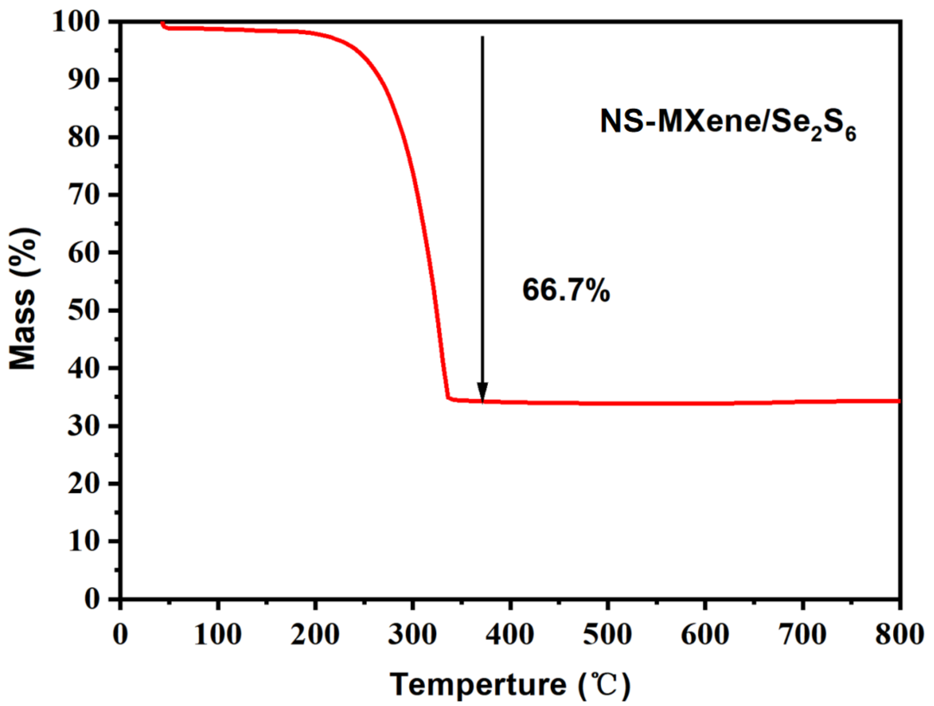

Figure S5. The TGA curve of NS-MXene/Se_2S_6.

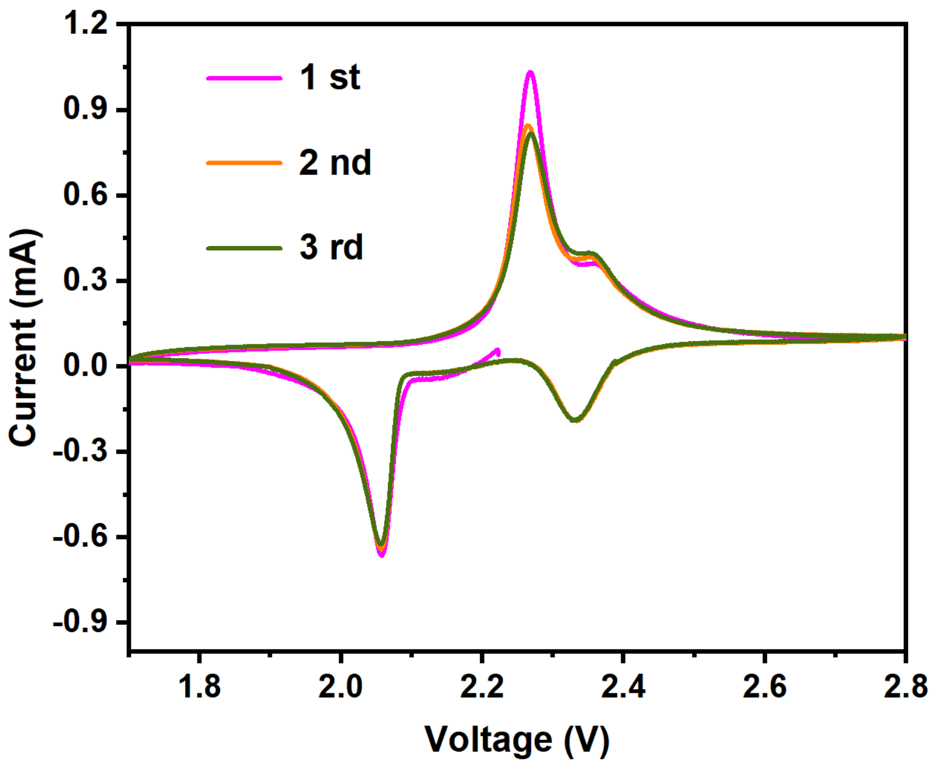

Figure S6. CV curves of cells with NS-MXene/S as cathodes at a scan rate of 0.1 mV s^{-1}.

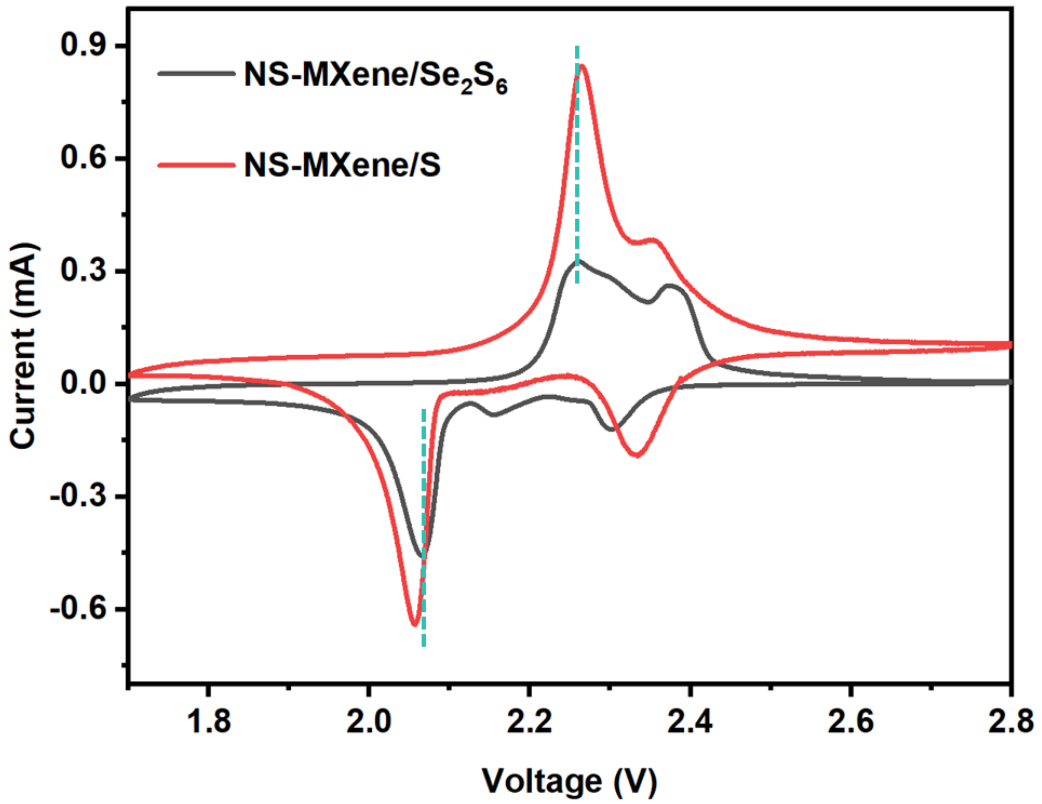

Figure S7. CV curves of cells with NS-MXene/S and NS-MXene/Se_2S_6 as cathodes for the second cycle at a scan rate of 0.1 mV s^{-1}.

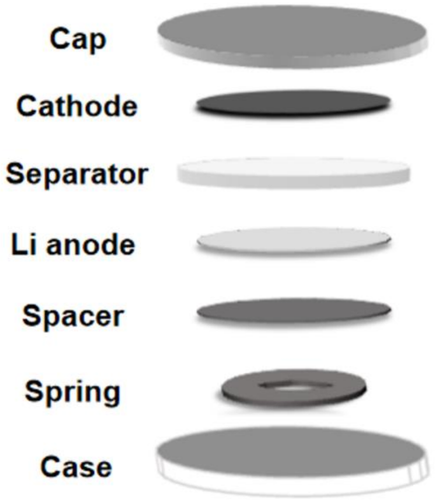

Figure S8. The structure diagram of Assembly cell.

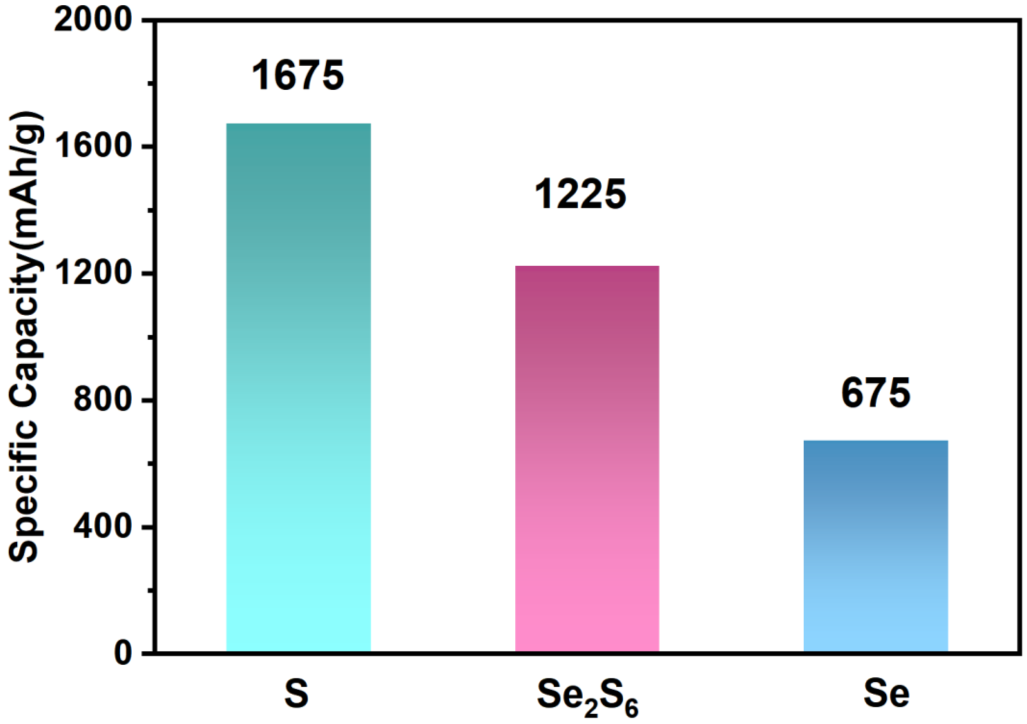

Figure S9. Theoretical specific capacity of the Se, S and Se_2S_6.

The electrode reaction between Li and Se_2S_6 molecules can be described as: $Se_2S_6 + 16Li^+ + 16e^- = 2Li_2Se + 6Li_2S$. Similarly, the electrode reaction between Li and S, Se molecules can be listed as: $S_8 + 16Li^+ + 16e^- = 8Li_2S$, $Se_8 + 16Li^+ + 16e^- = 8Li_2Se$. In addition, based on the Faraday's Law of electrolysis C = nF/M (C is the capacity of the electrochemical reaction, n is the number of electron transferred, F is Faraday's constant and equate to 96485 C mol^{-1}, M is molar mass of the active material), the theoretical capacity of S, Se andSe_2S_6 can be calculated as:

$C(S_8) = nF/M(S_8) = 16 * 96485\ C\ mol^{-1} / 256\ g\ mol^{-1} = 6030.3\ C\ g^{-1} = 1675\ mAh\ g^{-1}$

$C(Se_8) = nF/M(Se_8) = 16 * 96485\ C\ mol^{-1} / 632\ g\ mol^{-1} = 2442.6\ C\ g^{-1} = 675\ mAh\ g^{-1}$

$C(Se_2S_6) = nF/M(Se_2S_6) = 16 * 96485\ C\ mol^{-1} / 350\ g\ mol^{-1} = 4410.7\ C\ g^{-1} = 1225\ mAh\ g^{-1}$.

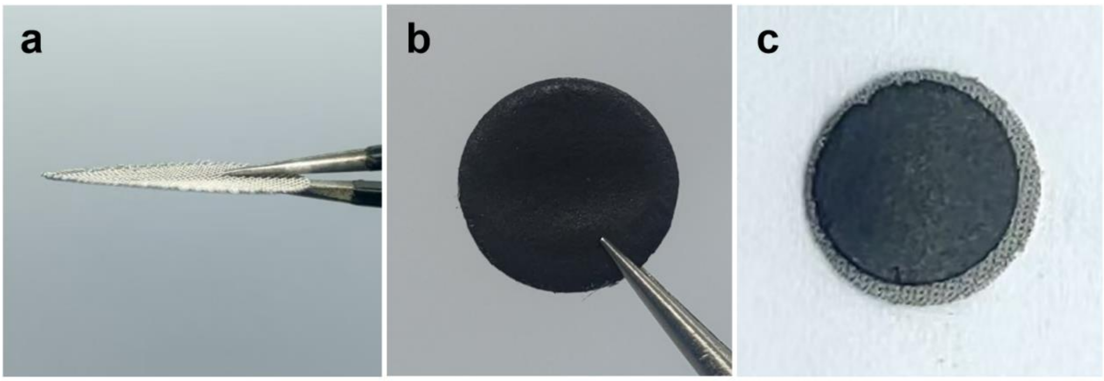

Figure S10. The digital photos of (a) Ti network (b) a self-standing NS-MXene/Se_2S_6 film (c) and Ti network-supported NS-MXene/Se_2S_6 electrode with high areal loading fabricated by mechanically pressing the NS-MXene/Se_2S_6 film onto current collector of Ti network.

.
